# Maternal corticosterone exposure in the mouse programs sex‐specific renal adaptations in the renin–angiotensin–aldosterone system in 6‐month offspring

**DOI:** 10.14814/phy2.12754

**Published:** 2016-04-27

**Authors:** James S. M. Cuffe, Danielle J. Burgess, Lee O'Sullivan, Reetu R. Singh, Karen M. Moritz

**Affiliations:** ^1^ School of Biomedical ScienceThe University of QueenslandSt LuciaAustralia

**Keywords:** fetal programming, kidney, RAS, sex specific

## Abstract

Short‐term maternal corticosterone (Cort) administration at mid‐gestation in the mouse reduces nephron number in both sexes while programming renal and cardiovascular dysfunction in 12‐month male but not female offspring. The renal renin–angiotensin–aldosterone system (RAAS), functions in a sexually dimorphic manner to regulate both renal and cardiovascular physiology. This study aimed to identify if there are sex‐specific differences in basal levels of the intrarenal RAAS and to determine the impact of maternal Cort exposure on the RAAS in male and female offspring at 6 months of age. While intrarenal renin concentrations were higher in untreated females compared to untreated males, renal angiotensin II concentrations were higher in males than females. Furthermore, basal plasma aldosterone concentrations were greater in females than males. Cort exposed male but not female offspring had reduced water intake and urine excretion. Cort exposure increased renal renin concentrations and elevated mRNA expression of *Ren1*,* Ace2,* and *Mas1* in male but not female offspring. In addition, male Cort exposed offspring had increased expression of the aldosterone receptor, *Nr3c2* and renal sodium transporters. In contrast, Cort exposure increased *Agtr1a* mRNA levels in female offspring only. This study demonstrates that maternal Cort exposure alters key regulators of renal function in a sex‐specific manner at 6 months of life. These finding likely contribute to the disease outcomes in male but not female offspring in later life and highlights the importance of renal factors other than nephron number in the programming of renal and cardiovascular disease.

## Introduction

Maternal stress is known to negatively impact fetal development and program long‐term health deficits for the adult offspring (Harris and Seckl 2011; Moisiadis and Matthews 2014). These programmed disease outcomes are often sex specific with cardiovascular and renal deficits being more prevalent or severe in males (Intapad et al. 2014). While sex hormones undoubtedly contribute to this sexual disparity, it is likely that programmed disease outcomes are at least partially attributable to differences in how male and female fetuses respond to a variety of different stresses (Barker et al. 2012). Maternal stress raises endogenous production of glucocorticoids, predominantly cortisol in the human or corticosterone (Cort) in the rodent. Studies in animal models have shown that maternal exposure to glucocorticoids during pregnancy can impair kidney development, reduce nephron endowment, and program the development of disease in adulthood (Moritz et al. 2005). However, these effects are dependent on the dose, timing, and type of glucocorticoid that the mother is exposed to and perhaps most importantly the sex of the developing fetus (Singh et al. 2012). Although there are many studies which have demonstrated an association between a nephron deficit and dysregulated blood pressure in adulthood, there is also significant evidence to suggest that a nephron deficit alone is unlikely to cause disease and that a number of additional renal factors are likely to be involved (Dorey et al. 2014).

We have previously reported that male and female mouse fetuses respond differently to maternal Cort exposure (33 *μ*g/kg/h) from E12.5 to E15 and demonstrated differences in growth and placental responses (Cuffe et al. 2012). Nephron number was reduced in both sexes but offspring physiological outcomes were sex specific. Surprisingly, while maternal Cort exposure resulted in male offspring developing albuminuria at 12 months of life, they were hypotensive (O'Sullivan et al. 2015). Following short‐term exposure to a saline challenge, male untreated and Cort‐exposed offspring had a small increase in mean arterial pressure and Cort‐exposed male offspring had elevated sodium excretion and fluid intake. In contrast, females had no overt physiological outcomes with or without the saline challenge (O'Sullivan et al. 2015). This suggests that Cort induces sex‐specific regulation of renal and cardiovascular outcomes despite similar nephron deficits.

The renin–angiotensin–aldosterone system (RAAS) plays a major role in the regulation of systemic blood pressure as well as renal sodium excretion and fluid homeostasis. While the RAAS acts a systemic cascade of interacting peptides and enzymes to regulate renal and cardiovascular function, the kidney is known to express all components of the renin–angiotensin–system (RAS) as well as key regulators of aldosterone signaling. Furthermore, the RAS is known to play a central role in the development of the fetal kidney. Our previous study demonstrated that maternal Cort exposure reduced the expression of renal *Agtr1a* and *Ren1* in male but not female fetuses (O'Sullivan et al. 2015) and supports previous work by our laboratory and others which demonstrate a key role of the RAS in regulating fetal responses to a maternal challenge (Moritz et al. 2010). While these alterations in utero may contribute to impaired kidney development we have not yet investigated how this system is affected in offspring. Other programming models have shown this system to be inhibited during development but upregulated in later life thereby contributing to cardiovascular and renal dysfunction (Moritz et al. 2010).

Aldosterone signaling in the kidney is mediated largely by the mineralocorticoid receptor (MR encoded by *Nr3c2*). Importantly glucocorticoids, including Cort can bind not only to the glucocorticoid receptor (GR – encoded by the gene *Nr3c1*) but also the MR (Reddy et al. 2009). Activation of the renal MR, normally through aldosterone binding, regulates transcription of a number of factors involved in sodium/potassium and fluid homeostasis including the sodium, potassium‐ATPase alpha 1 subunit (encoded by *Atp1a1*) as well as the epithelial sodium channels (ENaC subunits encoded by genes including Scnn1a and Scnn1b) (Viengchareun et al. 2007; Xanthakis and Vasan 2013). In adulthood, excess production of glucocorticoids or mineralocorticoids (Cicala and Mantero 2010) as well as altered renal *Nr3c2* signaling (Berger et al. 1998) impairs blood pressure regulation and renal function. Furthermore, given that circulating glucocorticoids are at much greater concentrations than plasma mineralocorticoids and can signal through Nr3c2, glucocorticoid metabolism by the hydroxysteroid 11 beta dehydrogenase enzymes (Hsd11b1 and Hsd11b2) plays an important role in regulating renal function. Thus, programmed alterations to this system following maternal glucocorticoid exposure, may contribute to impaired renal function and regulation of blood pressure in adult offspring.

Given the importance of the RAAS in the regulation of renal and cardiovascular function of the adult, this study aimed to investigate the effects of maternal Cort exposure on the RAAS in adult offspring. As hypotension and renal dysfunction were reported in male mice at 12 months of age (O'Sullivan et al. 2015), we sought to investigate renal function at 6 months of life to identify deficits which may precede the phenotype present in aged offspring. It was hypothesized that maternal Cort would induce sex‐specific dysregulation of factors involved in the renal RAAS and that these adaptations would be associated with signs of pathophysiology at 6 months of life.

## Materials and methods

### Animal treatment

All animal experimentation was approved by the University of Queensland animal ethics committee. C57BL/6 mice were time mated and either underwent surgical implantation of an osmotic minipump at embryonic day (E) 12.5 which delivered Cort for 60 h (33 *μ*g/kg/h) or were left untreated. Details of the animal model have been described in detail previously (Cuffe et al. 2011, 2012). Dams littered down and offspring were weaned at 21 days. At 6 months of age, renal function was assessed via urine collected from mice housed in metabolic cages for 24 hours (*n* = 6–8 from different litters). The animals were first acclimatized to the metabolic cage conditions by housing them in the cage for 4 h over two consecutive days. On the third day, the animal's body weight was recorded before it was placed into the cage. The amount of food and water offered to the animal was also recorded. After 24 h in the cage, the weight of the animal, and the amount of food and water left were again recorded. The volume of urine excreted was measured and the urine collected and frozen at −20°C for analysis. At the end of the 24‐h period, the animals were returned to their home cage. Animals were rapidly killed and blood collected for assessment of aldosterone levels and kidneys snap frozen in liquid nitrogen or fixed in 4% PFA for further analysis.

### Plasma hormone, renal RAS, and urinary electrolyte concentrations

Aldosterone levels were measured in heparinized plasma collected from 6‐month old male and female offspring (*n* = 6–8 per group) using a commercially available ELISA kit (Jomar Biosciences), as per the manufacturer's instructions. Renal tissue was carefully homogenized in radioimmunoassay buffer (*n* = 6–7 per group for males and *n* = 5 for females) and tissue renin as well as angiotensin II (Ang II), and angiotensin 1–7 (Ang 1–7) concentrations were measured by radioimmunoassay (Prosearch International) (Singh et al. 2013). Urine collected in metabolic cages over 24 h (*n* = 8–10 per group), was analyzed using the Cobas integra 400 Plus Chemistry Analyser to assess sodium, potassium, and chloride concentrations. Osmolality was measured in triplicate using a freezing point depression osmometer.

### mRNA and protein levels

RNA was extracted from whole kidneys (*n* = 6–8 per group, 1 animal/sex/litter) using RNeasy kits with on column DNase digestion (Qiagen). A quantity of 1 *μ*g of RNA was reverse transcribed using the iScript cDNA synthesis kit (BioRad) and 25 ng of cDNA was used per QPCR reaction as described previously. Commercially available Taqman assay of demand (AOD) primer/probe sets (life technologies) were used to measure mRNA levels of *Ren1* (Mm02342887_mh), *Atp6ap2* (Mm00510396_m1), *Ace* (Mm00802048_m1), *Ace2* (Mm01159003_m1), *Mas1* (Mm00434823_s1), *Agtr2* (Mm01341373_m1), *Nr3c1* (Mm00433832_m1), *Nr3c2* (Mm01241596_m1), *Hsd11b1* (Mm00476182_m1), *Hsd11b2* (Mm01251104_m1), *Atp1a1* (Mm00523255_m1), *Scnn1a* (Mm00803386_m1), *Scnn1b* (Mm00441215_m1), *Kim1* (*Havcr1*, Mm00506686_m1), *Il18* (Mm00434225_m1), *Fgf2* (Mm00433287_m1), *Col1a1* (Mm00801666_g1), and *Col1a2* (Mm00483888_m1) in kidneys collected from 6‐month old offspring. *Tgfb1* and *Agtr1a* mRNA levels were measured using custom‐made Taqman primer/FAM probe sets *Tgfb1* Forward Primer – TCGACATGGAGCTGGTGAAA, *Tgfb1* Probe – AAGCGCATCGAAGCCATCCGTG and *Tgfb1* Reverse Primer – GAGCCTTAGTTTGGACAGGATCTG; *Agtr1a* Forward Primer ‐GGGCTGTCTATACCGCTATGGAA, *Agtr1a* Probe‐ACCGCTGGCCCTTCGGCAA, and *Agtr1a* Reverse Primer‐ GCCGAAGCGATCTTACATAGGTG. All QPCR reactions were analyzed using the 2^−ΔΔCT^ method compared to the mean of two validated endogenous control genes (Rn18s and Actb) and normalized to the mean of the Untr group of their own sex.

Protein was extracted from 6‐month kidneys (*n* = 4–6 per group) using standard RIPA buffer for western blotting analysis of HSD11B2 and ACE2. A quantity of 20 *μ*g of total denatured protein was loaded per sample into a 12% polyacrylamide gel, transferred onto low fluorescence polyvinylidene membranes, and blocked using 4% fish gelatin in PBS. Membranes were incubated with rabbit primary antibody (HSD11B2‐1:1000, Cat. No. ab80317, Abcam, Melbourne, Australia, or ACE2‐1:333, Cat. No. Sc‐20998, Santa Cruz Biotechnologies, Santa Cruz, CA) diluted in blocking buffer overnight with a mouse beta actin loading control (Cat. No. A2228, Sigma Aldrich, 1 in 20000, Sydney, Australia) applied for 1 h before being incubated in anti‐mouse and anti‐rabbit fluorescent secondary antibodies for 1 h. Membranes were then scanned using the Licor Odyssey and densitometry was determined using the supplied software. Total protein levels of the proteins of interest were normalized to the beta actin loading control.

### Renal histopathology

Fixed kidneys were processed to paraffin and sectioned at 6 *μ*m before staining with hematoxylin and eosin for assessment of basic renal pathology.

### Statistical analysis

All data are presented as mean ± the standard error of the mean (SEM). Statistical analysis was performed using GraphPad Prism 6 for Windows. Sex differences were initially examined using unpaired *t*‐tests comparing Untr male values with Untr female values. Subsequent investigation into the effects of Cort on offspring outcomes used unpaired *t*‐tests compared with Untr values of the same sex. Litter averages were used to analyze body weight, kidney weight, and body to kidney weight ratios. For gene expression data, if more than two animals per litter were used in a group, data was analyzed using litter averages. Protein expression, tissue RAS levels, plasma aldosterone levels, and urine electrolyte levels were measured in one male and one female per litter when possible and analysis performed on individual animals. When data did not fit Gaussian distribution, nonparametric analyses were performed (Mann–Whitney test). If data had unequal variance, an unpaired *t*‐test with Welch's correction was applied. *P* < 0.05 was considered significant for all results.

## Results

### Body and kidney weights

At 6 months of life, male Untr offspring were heavier (*P* < 0.05) and had heavier kidneys than female Untr offspring (*P* < 0.05, Table 1). Maternal Cort exposure had no effect on offspring body weight, kidney weight, or kidney to body weight ratio in either male or female offspring (Table 1). The kidney to body weight ratio was similar between sexes regardless of treatment (*P* < 0.05, Table 1).

**Table 1:** Body weight, kidney weight, and urine electrolytes

	Male untr	Male cort	*P*‐value	Female untr	Female cort	*P*‐value
Body weight (g)	36.23 ± 0.98	35.28 ± 1.66	0.61	27.45 ± 0.97^a^	30.98 ± 1.63	0.08
Kidney weight (mg)	376.2 ± 22.6	388.2 ± 13.6	0.68	285.7 ± 17.8^a^	320.5 ± 11.8^a^	0.15
KW/BW ratio (mg/g)	10.38 ± 0.52	11.11 ± 0.63	0.38	10.54 ± 0.97	10.45 ± 0.67	0.94
Urinary sodium (mmol/L)	83.1 ± 9.6	98.5 ± 20.7	0.52	102.0 ± 34.7	106.7 ± 14.2	0.72
Sodium excretion (mmol/24 h/kg)	3.04 ± 0.48	1.93 ± 0.35	0.09	1.56 ± 0.36	2.63 ± 0.40	0.08
Urinary chloride (mmol/L)	103.9 ± 10.5	139.8 ± 33.1	0.34	129.0 ± 41.1	125.7 ± 14.4	0.62
Chloride excretion (mmol/24 h/kg)	3.89 ± 0.57	2.80 ± 0.62	0.23	2.08 ± 0.67	3.14 ± 0.43	0.19
Urinary potassium (mmol/L)	123.3 ± 16.4	165.7 ± 25.2	0.18	149.0 ± 44.4	117.0 ± 22.9	0.79
Potassium excretion (mmol/24 h/kg)	4.12 ± 0.49	3.42 ± 0.63	0.37	2.51 ± 0.76	3.37 ± 0.50	0.35

Untr‐ Untreated, Cort‐ Corticosterone. Data were analysed using unpaired *t* tests with or without Welch's correction or Mann–Whitney tests as required. *P*‐value given for the effect of Cort exposure compared to Untr of same sex.

a Represents *P* < 0.05 compared to male group of the same treatment. All data presented as mean ± SEM.

### Basal intrarenal RAS and plasma aldosterone concentrations in males and females

**Figure 1:**
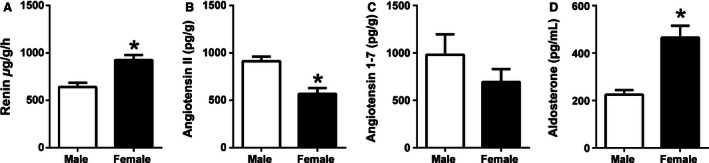
Basal renal renin concentrations (A), renal angiotensin II concentrations (B), renal angiotensin 1–7 concentrations (C), and plasma aldosterone concentrations (D) in male (open bar) and female (closed bar) mice at 6 months of life. Data are presented as means ± SEM,* n* = 6–8 per group. *P < 0.05,

Intrarenal renin concentrations were found to be 44% higher in Untr females compared to Untr males (*P* < 0.05, Fig. 1A), whereas renal Ang II concentrations were 38% lower in Untr females compared to Untr males (*P* < 0.05, Fig. 1B). In contrast, Ang 1–7 concentrations were similar between Untr males and Untr females (Fig. 1C). Plasma aldosterone concentrations in Untr female offspring were more than twofold higher than Untr male offspring (*P* < 0.05, Fig. 1D).

### Effect of prenatal Cort on renal RAS and plasma aldosterone concentrations

**Figure 2:**
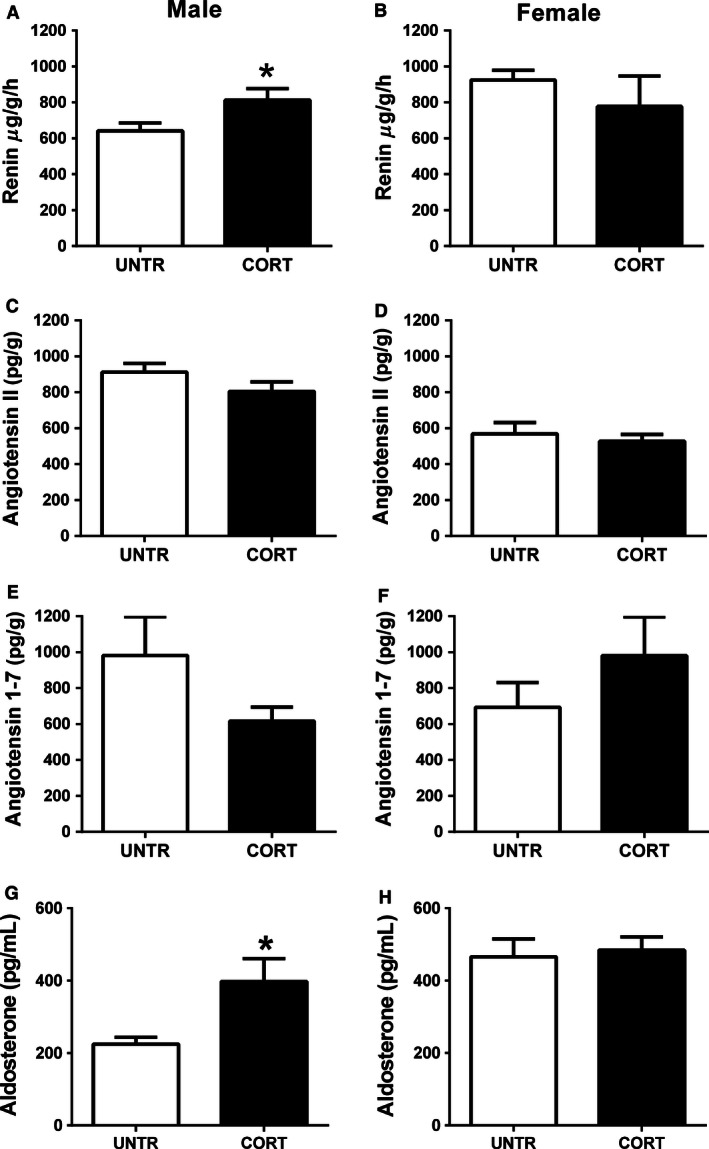
The effects of maternal corticosterone (Cort) exposure (closed bars) from E12.5 to E14.5 on renal renin concentrations (A‐male, B‐female), Angiotensin II levels (C‐male, D‐female), Angiotensin 1–7 levels (E‐ male, F‐female), and plasma Aldosterone levels (G‐male and H‐female) at 6 months of life compared to untreated controls (open bars). Data are presented as means ± SEM,* n* = 6–8 per group. *P < 0.05.

Tissue renal renin concentrations were increased in males (*P* < 0.05, Fig. 2A) but not in females (Fig. 2B), prenatally exposed to Cort. Renal tissue Ang II as well as Ang 1–7 concentrations were not affected by prenatal exposure to maternal Cort in either male or female offspring (Fig. 2C–F). Plasma aldosterone concentrations were 77% higher in Cort‐exposed male offspring compared to Untr controls (*P* < 0.05, Fig. 2G). Plasma aldosterone levels were similar in both Cort and Untr female offspring (Fig. 2H).

### Food and water consumption and renal physiology at 6 months

**Figure 3:**
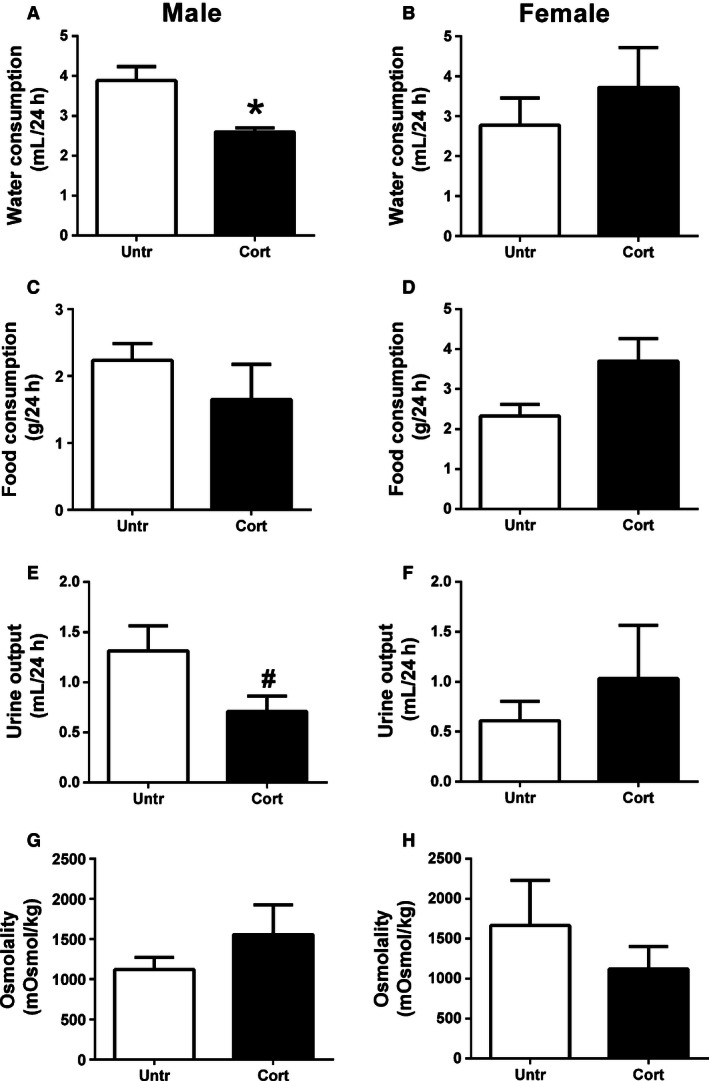
The effects of maternal corticosterone (Cort) exposure (closed bars) from E12.5 to E14.5 on water consumption (A‐male, B‐female), food consumption, (C‐male, D‐female), urine output (E‐ male, F‐female), and urine osmolality (G‐male and H‐female) at 6 months of life compared to untreated controls (open bars). Data are presented as means ± SEM,* n* = 6–8 per group. *P < 0.05. ^#^ *P* = 0.06.

Basal water consumption, food consumption, urine output, and osmolality were not statistically different between male and female offspring (Fig. 3). Prenatal Cort exposure, however, reduced water consumption in male (*P* < 0.05, Fig. 3A) but not female offspring (Fig. 3B) compared to Untr controls. Prenatal Cort treatment had no impact on food intake (Fig. 3C and D) or urine osmolality (Fig. 3G and H) in either males or females. While urine output was decreased by 45% in Cort‐exposed male offspring compared to Untr male offspring, this did not reach statistical significance (*P* = 0.06, Fig. 3E). Urine excretion rate was not affected by prenatal treatment in female offspring (Fig. 3F). Urine sodium, chloride, and potassium concentrations and excretion rates were not different between the Cort and Untr groups regardless of sex (Table 1).

### Renal mRNA and protein levels in adult life following prenatal Cort exposure

**Figure 4:**
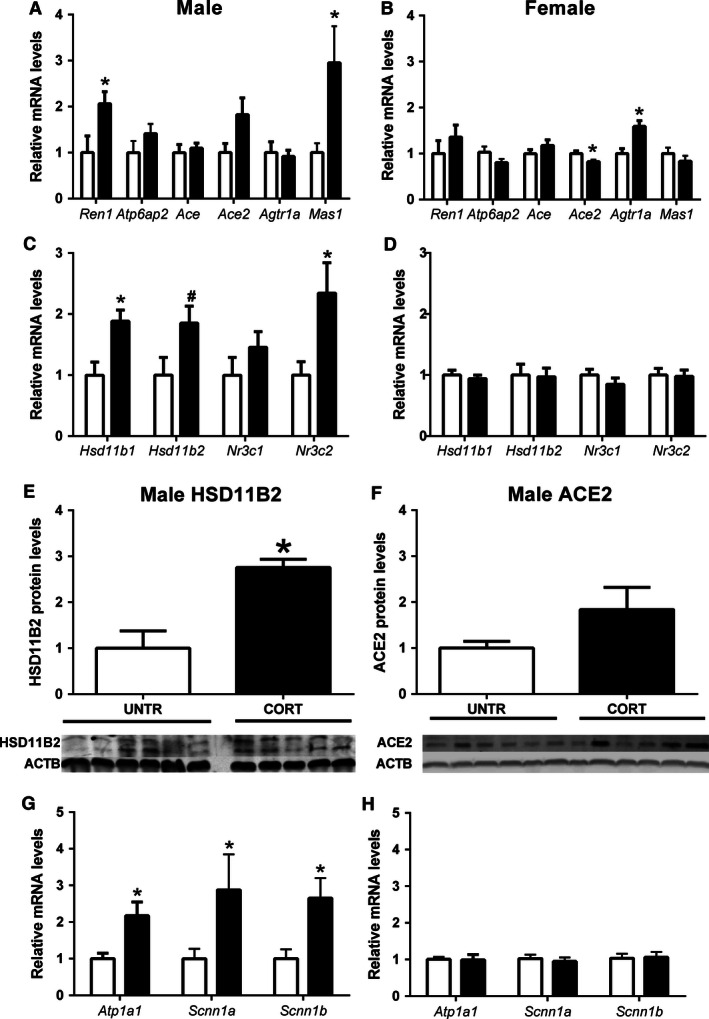
The effects of maternal corticosterone (Cort) exposure (closed bars) from E12.5 to E14.5 on: expression of genes related to the renal renin–angiotensin system (A‐male, B‐female) and renal mineralocorticoid/glucocorticoid sensitivity (C‐male, D‐female), renal HSD11B2 protein expression (E) and ACE2 protein expression (F), and renal sodium transporter mRNA levels (G‐male, H‐female) in offspring at 6 months of life. Data are presented as means ± SEM,* n* = 8–10 per group for gene expression and 4–6 per group for protein expression. *P < 0.05, ^#^ *P* = 0.06.

Prenatal exposure to Cort significantly increased the renal expression of *Ren1* (*P* < 0.05) and *Mas1* (*P* < 0.05) and resulted in a trend (*P* = 0.08) for increased *Ace2* mRNA levels in 6‐month old male offspring (Fig. 4A). ACE2 protein levels were not significantly affected by prenatal treatment in male (*P* = 0.12, Fig. 4F) or female offspring (Untr 1.00 ± 0.10, Cort 1.17 ± 0.20). *Atp6ap2*,* Ace*, and *Agtr1a* mRNA levels in male kidneys were not affected by prenatal Cort exposure. In females, *Ace2* mRNA levels (*P* < 0.05) were reduced while *Agtr1a* levels (*P* < 0.05) were increased by prenatal Cort exposure. *Ren1*,* Atp6ap2*,* Ace*, and *Mas1* were all unaffected in female offspring prenatally exposed to Cort (Fig. 4B).

Maternal Cort exposure resulted in significant upregulation of *Hsd11b1* (*P* < 0.05) and *Nr3c2* (*P* < 0.05) mRNA levels in 6‐month male offspring. In addition, mRNA expression of *Hsd11b2* tended to be increased (*P* = 0.06) (Fig. 4C) while the HSD11B2 protein levels were increased significantly in male offspring following prenatal Cort exposure (*P* < 0.05, Fig. 4E). In contrast, in kidneys of females, mRNA expression of *Hsd11b1*,* Hsd11b2*,* Nr3c1*, and *Nr3c2* (Fig. 4D) as well as HSD11B2 protein expression (data not shown) were not affected by prenatal Cort exposure. Male offspring of Cort exposed dams were found to have increased renal mRNA levels of *Atp1a1* (*P* < 0.05), *Scnn1a* (*P* < 0.05), and *Scnn1b* (*P* < 0.05, Fig. 4G). In females, kidney mRNA levels of *Atp1a1, Scnn1a*, and *Scnn1b* were not affected by prenatal treatment (Fig. 4H).

### Markers of renal fibrosis

**Figure 5:**
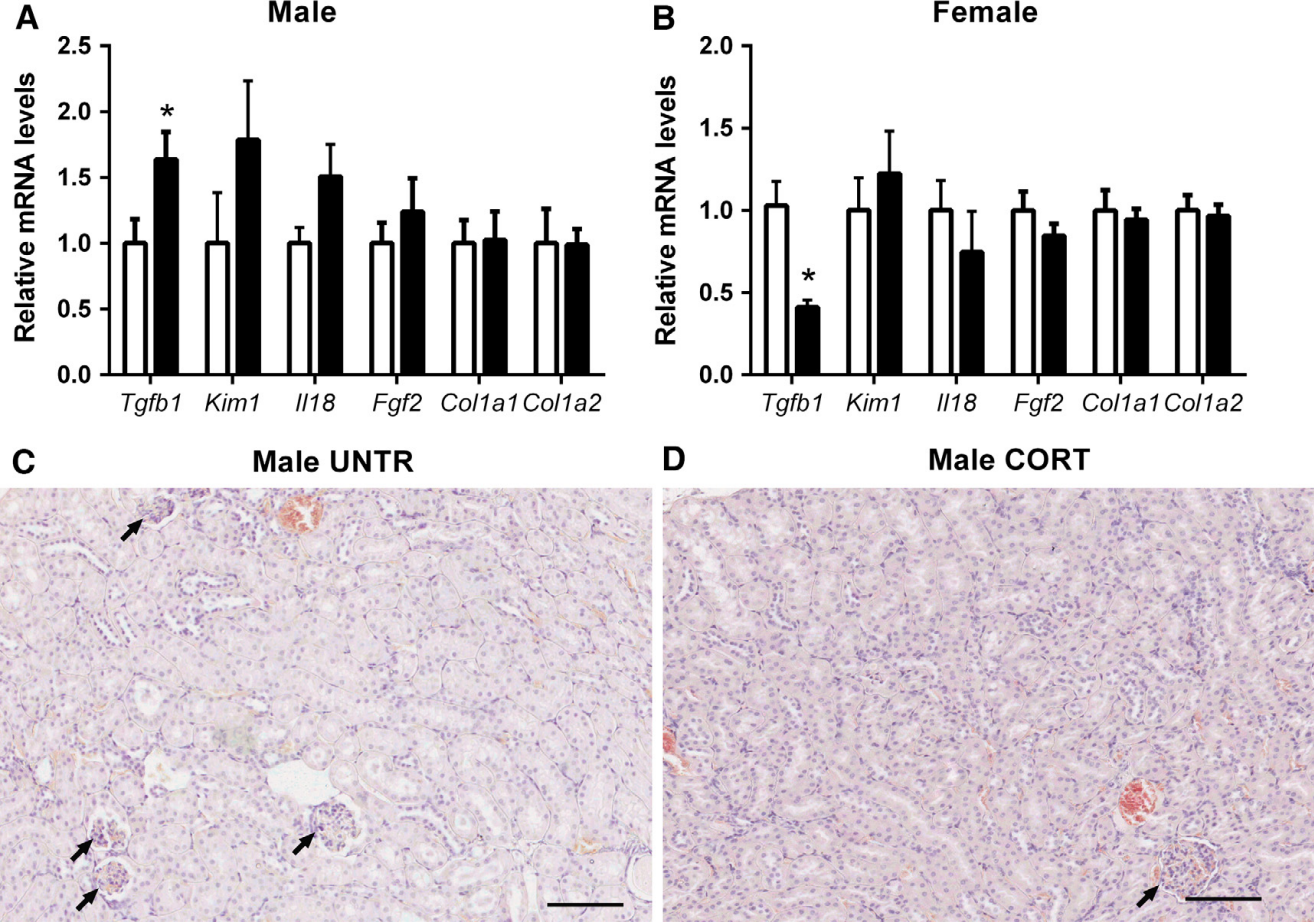
The effects of maternal corticosterone (Cort) exposure (closed bars) from E12.5 to E14.5 on mRNA expression of markers of renal fibrosis/pathology in male (A) and female (B) offspring at 6 months of life compared to untreated controls (open bars). Renal histopathology in Untr (C) and Cort exposed (D) male offspring at 6 months of life. Data are presented as means ± SEM,* n* = 8–10 per group. *P < 0.05, Arrows point to Glomeruli.

Given the programmed changes in renal function at 12 months of age, we investigated the mRNA expression of markers of renal fibrosis at 6 months to determine if early changes contributing to renal pathology could be detected. *Tgfb1* expression was 63% higher in male offspring prenatally exposed to Cort compared to Untr controls (*P* < 0.05) (Fig. 5A). In contrast, *Tgfb1* expression was decreased by 59% in female Cort‐exposed offspring (*P* < 0.05) (Fig. 5B). mRNA levels of *Kim1*,* Il18*,* Fgf2*,* Col1a1*, and *Col1a2* were not affected by prenatal treatment in either males or females. Histological examination demonstrated no overt signs of renal pathology or fibrosis (Fig. 5C and D).

## Discussion

We have recently reported that maternal Cort exposure in the mouse reduces nephron number in both sexes but programs dysregulated renal function and blood pressure in male offspring only at 12 months of age (O'Sullivan et al. 2015). Given the importance of the RAAS in the regulation of renal physiology and blood pressure, this study investigated whether maternal Cort dysregulated this system in a sex‐specific manner. This study demonstrates for the first time in the mouse that maternal Cort exposure programs an increase in renal renin concentrations and *Ren1* mRNA expression in male but not female offspring and this is associated with increased mRNA expression of additional key components of the renal RAS. Prenatal Cort exposure similarly dysregulates offspring plasma aldosterone levels and programmed elevations in *Nr3c2*, (the mineralocorticoid receptor) while increasing expression of *Hsd11b1* and *Hsd11b2*, enzymes involved the regulation of glucocorticoid‐induced *Nr3c2* signaling. Collectively, this increase in aldosterone in combination with elevated levels of *Nr3c2* may contribute to the increased expression of aldosterone‐regulated renal sodium transporters in male offspring. Interestingly, female offspring appear largely unaffected with only small changes in *Agtr1a* and *Ace2* mRNA levels. These findings indicate that prenatal Cort exposure during kidney formation programs alterations in key components of the RAAS in a sex‐specific manner. It would appear, however, that compensatory adaptations maintain a physiological balance which prevents overt dysfunction or pathology at 6 months. However, the dysregulated RAAS at this age may contribute to the programmed renal and cardiovascular pathologies that occurs with aging (O'Sullivan et al. 2015).

### The effect of sex on plasma aldosterone and the renal RAS

We have previously demonstrated different expression profiles for key components of the RAAS in placentas of male and female mice (Cuffe et al. 2014) and demonstrated that maternal glucocorticoid exposure can induce sex‐specific differences in fetal kidney RAAS expression (O'Sullivan et al. 2015). However, to understand the role of the RAAS in the sex‐specific regulation of programmed disease in the mouse, it was important that basal RAAS levels were determined in adult offspring (Reckelhoff 2001). Plasma renin activity is frequently reported as being higher in males than females, (Komukai et al. 2010) however, this likely reflects differences in substrate levels as opposed to levels of renin in the kidney. Human, and most animal studies report plasma renin activity rather than tissue concentrations. Pendergrass et al. demonstrated in the Lewis rat that while plasma renin concentrations tend to be higher in males than females, renal renin concentrations are greater in females than males (Pendergrass et al. 2008). Surprisingly, despite renin concentrations being higher in females than males in our mouse model, Ang II levels were higher in males than females and Ang 1–7 levels were similar between sexes. While concentrations of renal angiotensin peptides have been shown to be similar in males and females in Lewis rats (Pendergrass et al. 2008), less is known about basal sex differences in mice. This study demonstrated that female mice have higher levels of aldosterone than male mice at 6 months of age. Interestingly, aldosterone drives upregulation of *Ren1* gene expression in juxtaglomerular cells (Klar et al. 2004) which may provide a mechanism for the sex‐specific renal renin concentrations currently reported. Similarly in the rat, plasma aldosterone concentrations are frequently reported as being higher in females than males (Martin et al. 1987). Studies in humans have provided conflicting data regarding sex differences in plasma aldosterone concentrations with some studies suggesting aldosterone concentrations are higher in women than men (Clark et al. 1990; Hlavacova et al. 2013), but others reporting lower concentrations (Miller et al. 1999). Additional studies have demonstrated that although women tend to have higher plasma aldosterone levels than men, following salt loading, women have lower values than men (Harris and Seckl 2011). The currently described basal differences in RAAS concentrations with males having an Ang II‐dominant RAAS and females having an aldosterone‐dominant RAAS, may infer different disease risks and may contribute to the sex differences in disease outcomes in models of programmed disease.

### The effect of prenatal Cort exposure on offspring RAAS and physiology

Numerous studies have demonstrated that prenatal perturbations including natural glucocorticoids induce long‐term dysregulation of the RAAS (Moritz et al. 2002, 2010; Singh et al. 2007). Importantly, here we report that programmed changes to the RAAS are sexually dimorphic. Male offspring of Cort‐exposed dams demonstrated increased tissue renin concentrations in association with increased renal *Ren1*,* Mas1,* and *Ace2* mRNA expression. While an increase in renal renin concentrations would be expected to increase renal Ang II content, we observed no change in renal Ang II following prenatal Cort exposure. Given that *Ren1* mRNA levels and renin tissue concentrations are both increased and yet Ang II levels are unchanged, this suggests an increase in cleavage of Ang I and/or Ang II into smaller peptides. There was a tendency for increased *Ace2* expression in male offspring following prenatal Cort exposure which can cleave Ang I into Ang 1–9 and Ang II into Ang 1–7. While we demonstrated that renal Ang 1–7 levels were not different, we have not measured renal levels of other cleaved angiotensin products such as Ang 1–9 (Ocaranza et al. 2014) or Ang IV (Ferrao et al. 2014). Although *Agtr1a* mRNA levels were not affected in male offspring prenatally exposed to Cort, *Mas1* mRNA levels were elevated ~ threefold. Mas1 is the primary receptor for Ang 1–7 and is thought to mediate the antihypertensive effects of Ang 1–7. Mas1 null mice have reduced urine volume and fractional sodium excretion suggesting a key role for Mas in regulating renal function (Pinheiro et al. 2009). In a model of betamethasone exposure known to increase blood pressure in offspring, Mas1 expression was reduced in the dorsal medulla (Marshall et al. 2013). Collectively, the effects of prenatal Cort exposure on the renal RAS of male offspring may be predictive of increased sodium retention, reduced water excretion and increased blood pressure. While we present data from this study indicating reduced water excretion, increased blood pressure does not eventuate in this model possibly at least partially due to the compensatory reduction in fluid intake (O'Sullivan et al. 2015). It is interesting to note that both renin and prorenin stimulate fibrosis through production of fibrotic factors including Tgfb independently of Ang II by signaling though the prorenin receptor (Clavreul et al. 2011). Therefore, the increased renin, may be acting to stimulate fibrosis rather than to increase Ang II‐regulated renal function. Indeed at 6 months of age, while there was no overt histological pathology, *Tgfb1* expression was increased. However, downstream fibrotic markers (i.e., *Col1a1*,* Col1a2*) were unaffected (Wu et al. 2013).

Following maternal Cort exposure, plasma aldosterone levels were elevated in male but not female offspring. This increase in aldosterone concentrations may be of great significance considering the almost threefold increase in mRNA levels of mineralocorticoid receptor (*Nr3c2*). Aldosterone production is regulated predominantly by plasma Ang II or ACTH. Indeed, while renal Ang II levels were unchanged in this study, plasma Ang II levels were not assessed and may be contributing to the phenotype observed. Furthermore, it is likely that the increased production of aldosterone is regulated at least partially by programmed changes to the HPA as have been reported previously following maternal glucocorticoid administration (Waddell et al. 2010). In addition to the increase in plasma aldosterone concentrations, we demonstrate that protein levels of HSD11B2 and mRNA levels of *Hsd11b1* are both increased in Cort‐exposed male offspring. The predominant isoform of Hsd11b in the kidney is Hsd11b2, and while Hsd11b1 may be acting to oppose the actions of Hsd11b2 in the kidney, studies have suggested that both isoforms of this enzyme may contribute to the metabolism of glucocorticoids in the kidney (Gong et al. 2008), thus reducing the impact of circulating glucocorticoids on renal function. It is important to note that it is difficult to draw conclusions based on mRNA and protein levels of these enzymes and although others have demonstrated that gene expression closely correlates with enzyme activity at least in rodent kidneys (Liu et al. 1998), enzyme activity data would be highly informative.

Aldosterone acts on the mineralocorticoid receptors to increase sodium and water reabsorption and increase blood pressure (Goodwin and Geller 2012; Xanthakis and Vasan 2013). Indeed we demonstrated that prenatal Cort exposure increased *Atp1a1* as well as both *Scnn1a* and *Scnn1b* in male offspring only. Scnn1a and Scnn1b encode the alpha and beta subunits of the ENaC sodium channels which are located on the apical membrane of the principle cells of the collecting duct and regulate sodium reabsorption from the tubular lumen. *Atp1a1* encodes the alpha subunit of the Na^+^/K^+^‐ATPase pump which acts at the basolateral membrane of the collecting duct cells to clear out the reabsorbed sodium from within the tubular cell and into the renal interstitial fluid (Lang et al. 2005). This alters the sodium gradient and additionally results in increased water reabsorption. While we have not directly measured the activity of these transporters, it is likely that the Cort induced increase in their expression has contributed to the reduced urine output observed. We have previously demonstrated in the sheep that maternal cortisol exposure increases the expression of *Scnn1a*,* Scnn1b*, and *Scnn1g* as well as *Atp1a1* and *Atp1b1* during fetal life but only increases in *Atp1a1* in offspring (Moritz et al. 2011). While sex‐specific differences were not measured in the sheep model, this suggests that alterations in sodium channel expression in this study may have originated in utero. In addition to the reduction in urine output, water intake was found to be decreased. This highlights that although this study has focused on renal outcomes, maternal Cort exposure likely has long‐term programming effects on nonrenal regulators of offspring physiology. The reduction in water intake demonstrated in this study may be indicative of reduced thirst. As reduced thirst can lead to hypovolemia and reduced blood pressure, the reduced water intake is likely to be of significance and may contribute to the phenotypes observed in later life. A number of studies have demonstrated that regulation of offspring thirst can be programmed by prenatal insults (Mecawi et al. 2015), however, many of these studies report increased thirst rather than reduced thirst. Offspring of rats exposed to a low‐protein diet during pregnancy were found to drink more and excrete more urine (Alwasel et al. 2012). These rats were found to have an increased drive for salt intake and increased extra cellular fluid volume in association with increased blood pressure. It is interesting to note that rats exposed to a low‐protein diet that later developed increased blood pressure were found to have a suppressed renal RAAS at birth (Woods et al. 2001). In a rat model of prenatal nicotine exposure, female but not male offspring were found to have reduced water intake after a water deprivation challenge compared to controls (Hui et al. 2009). This was associated with reduced levels of Agtr1 and Agtr2 protein in the forebrain of nicotine‐exposed female offspring. Increased plasma Ang II or changes in neurological RAAS signaling can both drive increased thirst, whereas atrial natriuretic peptide (ANP) which is produced in the heart in response to increased plasma Ang II or plasma sodium, reduces thirst (McKinley and Johnson 2004). While renal levels of Ang II were not affected by prenatal treatment in this study, it is possible that plasma Ang II levels or RAS receptors in the brain may have been affected and could have contributed to the reduction in thirst in this study.

Interestingly, all other markers of renal function were unaffected by prenatal treatment at 6 months of life and although we have not measured blood pressure at this age, prenatal Cort exposure results in significant and sustained hypotension, rather than hypertension in male offspring at 12 months. This study demonstrates hypertensive‐like phenotypes related to aldosterone signaling while the changes in RAS levels tips this phenotype toward increased sodium excretion and hypotension. It is tempting to speculate that with aging, this may contribute toward the phenotypes observed at 12 month of age.

Importantly, this study demonstrated that none of the Cort induced changes in RAS expression observed in males were present in females. In fact, while the protective arm of the RAS was programmed to be increased in male offspring, female offspring had a small but significant increase in *Agtr1a* gene expression and reductions in *Ace2* gene expression. This would be indicative of increased Ang II sensitivity as not only would Ang II have greater receptor availability but Ace2 would also break it down less readily. However, no change in renal renin, Ang II, or Ang1–7 were observed in the female Cort‐exposed offspring. Given that female offspring have no overt phenotype either in this study (6 months) or at 12 months (O'Sullivan et al. 2015), these small changes in renal RAS mRNA levels in female offspring appear to be of little functional consequence.

## Conclusion

This study demonstrates that maternal Cort exposure can dysregulate the renal RAAS and induce minor impairments in renal physiology in male but not in female offspring. These minor renal impairments precede the onset of renal dysfunction at 12 months of age but provide insight into the sex‐specific dysregulation of renal/cardiovascular physiology and highlights that the adaptive capacity of the kidney is reduced with aging (Yang and Fogo 2010). While it is likely that these sex‐specific phenotypes were programmed in utero, clear differences in male and female RAAS levels likely contributed to the development of disease. This study demonstrates the complex role of the kidney and highlights the renal RAAS as being central in programmed hypotension, although these findings must be considered in the context of whole‐animal physiology in which multiple organs and systems are likely to be involved.

## Conflict of Interest

None declared.
